# Artificial Intelligence in Pharmaceutical Technology and Drug Delivery Design

**DOI:** 10.3390/pharmaceutics15071916

**Published:** 2023-07-10

**Authors:** Lalitkumar K. Vora, Amol D. Gholap, Keshava Jetha, Raghu Raj Singh Thakur, Hetvi K. Solanki, Vivek P. Chavda

**Affiliations:** 1 School of Pharmacy, Queen’s University Belfast, 97 Lisburn Road, Belfast BT9 7BL, UK; r.thakur@qub.ac.uk; 2 Department of Pharmaceutics, St. John Institute of Pharmacy and Research, Palghar 401404, Maharashtra, India; amolg@sjipr.edu.in; 3 Department of Pharmaceutics and Pharmaceutical Technology, L. M. College of Pharmacy, Ahmedabad 380009, Gujarat, India; keshavjetha@gmail.com; 4 Ph.D. Section, Gujarat Technological University, Ahmedabad 382424, Gujarat, India; 5 Pharmacy Section, L. M. College of Pharmacy, Ahmedabad 380009, Gujarat, India

**Keywords:** artificial intelligence (AI), machine learning, drug discovery, formulation, dosage form testing, pharmacokinetics, pharmacodynamics, PBPK, QSAR

## Abstract

Artificial intelligence (AI) has emerged as a powerful tool that harnesses anthropomorphic knowledge and provides expedited solutions to complex challenges. Remarkable advancements in AI technology and machine learning present a transformative opportunity in the drug discovery, formulation, and testing of pharmaceutical dosage forms. By utilizing AI algorithms that analyze extensive biological data, including genomics and proteomics, researchers can identify disease-associated targets and predict their interactions with potential drug candidates. This enables a more efficient and targeted approach to drug discovery, thereby increasing the likelihood of successful drug approvals. Furthermore, AI can contribute to reducing development costs by optimizing research and development processes. Machine learning algorithms assist in experimental design and can predict the pharmacokinetics and toxicity of drug candidates. This capability enables the prioritization and optimization of lead compounds, reducing the need for extensive and costly animal testing. Personalized medicine approaches can be facilitated through AI algorithms that analyze real-world patient data, leading to more effective treatment outcomes and improved patient adherence. This comprehensive review explores the wide-ranging applications of AI in drug discovery, drug delivery dosage form designs, process optimization, testing, and pharmacokinetics/pharmacodynamics (PK/PD) studies. This review provides an overview of various AI-based approaches utilized in pharmaceutical technology, highlighting their benefits and drawbacks. Nevertheless, the continued investment in and exploration of AI in the pharmaceutical industry offer exciting prospects for enhancing drug development processes and patient care.

## 1. Introduction

Numerous industries are striving to enhance their progress to meet the demands and expectations of their customers, utilizing various methodologies. The pharmaceutical industry is a critical field that plays a vital role in saving lives. It operates based on continuous innovation and the adoption of new technologies to address global healthcare challenges and respond to medical emergencies, such as the recent pandemic [1]. In the pharmaceutical industry, innovation is typically predicated on extensive research and development across various domains, including but not limited to manufacturing technology, packaging considerations, and customer-oriented marketing strategies [2]. Novel pharmaceutical innovations are range from small drug molecules to biologics, with a preference for better stability with high potency to fulfil unmet needs to treat diseases. The assessment of the significant levels of toxicity associated with new drugs is an area of considerable concern, necessitating extensive research and exploration in the foreseeable future. One of the primary aims is to provide drug molecules that offer optimal benefits and suitability for utilization in the healthcare industry. Despite this, the pharmacy industry faces numerous obstacles that necessitate further advancement using technology-driven methods to address worldwide medical and healthcare demands [3,4,5].

The need for a proficient workforce in the healthcare industry is persistent, necessitating the continuous provision of training to healthcare personnel to augment their involvement in routine duties. Identifying skill gaps in the workplace is a crucial undertaking within the pharmaceutical industry. It is imperative to effectively address the identified gaps through appropriate remedial measures while acknowledging that providing adequate training can also pose a significant challenge. As per a report presented by certain authorities, it has been observed that approximately 41% of supply chain disruptions occurred in June 2022. The report further highlights that supply chain disruption has emerged as the second-most-formidable challenge to overcome. Several pharmaceutical industries are anticipating further advancements in their supply chain, as well as innovative models to address these challenges, with the potential to enhance business resilience [6]. The global outbreak of coronavirus disease 2019 (COVID-19) has caused significant disruptions to various operations worldwide, including ongoing clinical trials [7].

Pandemics, natural catastrophes, pricing changes, cyberattacks, logistical delays, and product issues increase supply chain disruptions. Transportation challenges caused by the epidemic have devastated the supply chain network and global industries. Decision-induced delays for price updates from suppliers owing to misunderstanding over whether to utilize the new price or the existing price for commodities or materials create price fluctuation delays. New obstacles arise from countries’ cross-border trade cooperation strategies, increasing criminal activity and instability in the availability of crucial resources for operation and production. The manufacturing of footprint modifications is needed to suit patient needs and compliance.

Within the pharmaceutical industry, a significant quantity of COVID-19 vaccines ended up being unusable during the pandemic because of complications related to the maintenance of the cold chain. The primary cause of supply chain disruption resulting from the delayed response can be attributed to insufficient innovation and imprecise forecasting in industrial and commercial operations. Supply chain disruptions within the pharmaceutical industry have significant ramifications on customer satisfaction, corporate reputation, and potential profits [8,9].

**Figure 1:**
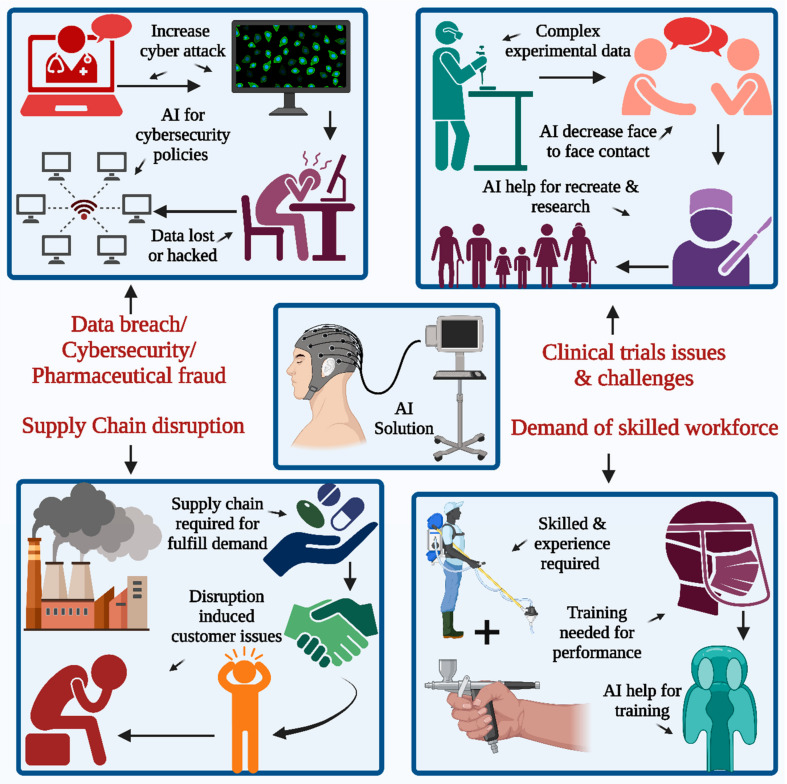
Depicts a possible artificial intelligence (AI) solution to the pharmaceutical industry’s challenges: acquiring a proficient workforce is a prerequisite in all sectors to leverage their expertise, proficiency, and aptitude in product innovation. The second pertains to supply chain disruption and clinical trial experimentation challenges. The incidence of cyberattacks is on the rise, with data breaches and security emerging as significant concerns for the industry.

The implementation of AI is poised to bring about a significant transformation in the way the pharmaceutical industry handles supply chain operations (Figure 1). It also consolidates numerous AI research endeavors from recent decades to create effective solutions for diverse supply chain issues. Additionally, the study suggests potential research areas that could enhance decision-making tools for supply chain management in the future [10,11].

The primary impact of the pandemic is receding, but it still has some influence on clinical trials. Many pharmaceutical companies are looking to adopt newer technologies, including platforms such as AI and virtual platforms in this field. These new technologies may be helpful in the restart or recreation of these clinical trials, with minimal interaction for face-to-face types [12,13,14,15,16,17,18], as presented in Figure 1. At present, highly skilled workers and high maintenance costs pose a larger challenge. The fourth main challenge in seeking a technology-based solution is data breaches and cybersecurity threats. The number of cyberattacks on available patient data has also increased in the 21st century, and many pharmaceutical companies are more concerned about confidential medical records and patient data, which are especially vulnerable to cybersecurity attacks. Some of the major challenges associated with traditional clinical trials are data fragmentation and disconnected system involvement, which generally result from scattered data generated during the trials and hence require extensive manual data transcription efforts for documents along with those of the systems. There is a lack of innovation in the trial models, which thus requires the rework and repetition of the ongoing work. In the healthcare sector, patient recruitment, enrollment, monitoring, retention, and medical adherence are the key points that require special attention due to clinical trials. The enrollment of the patient is affected due to the traveling process at the trial sites, which is time-consuming for the participants, and frequent visits to sites contribute to patient re-enrollment in the same context. The application of AI to the study design helps with optimization as well as accretion for the work related to the creation of the patient-centric type of design. AI uses techniques for the collection of the huge amounts of data generated from those clinical trials, thus reducing the amount of data manpower required for the same. Such technologies implement body sensors along with wearable devices to record the patient’s vital signs and valuable information in a remote mode, which helps meet the patient’s requirement for face-to-face interaction on a routine basis. AI algorithms using wearable technology provide real-time insights during the study process [19].

A new technology platform and solution are required for the implementation of effective cybersecurity inside the office and for remote workers. Special attention must also be paid to data security and breach techniques. Technology is also required to address political fraud, and many cases have been reported, especially during the pandemic in the last few years around the world. Therefore, there is a need to take appropriate steps for the prevention of healthcare fraud, along with constant encouragement for internal discussions about fraudulent behaviors, which may help in the inhibition of the same. 

## 2. Current Pharmaceutical Challenges and the Role of AI

In the pharmaceutical industry, research on small molecules for better products and customer satisfaction is ongoing due to their multiple advantages. The chemical synthesis process is simple, while the synthetic derivative preparation is economical. Thus, many stable and potent small-molecule-loaded formulations are present in the pharmacy sector. Except for the treatment of rare diseases, many innovative small molecules face competition from generic molecules, and complex data are required for them to be launched, along with clinical trials. These processes increase the economic pressure on companies to engage in more innovation. However, the biomolecular drug industry is still growing at a rapid pace to compensate for the crisis induced by the small molecular size and poor dissemination of research and innovations. Small-molecule actions are based on their conformation and reactivity [20,21,22,23,24,25,26]. Biomolecules, which are large units, mostly contain amino acids from the protein source along with nucleotides or ribonucleotides for the nucleic acid. Their stability and function are also influenced by the supramolecular sequence and the spatial conformation [27]. Some biomolecules are very successful products, such as insulin and adalimumab. The pharmacokinetic aspects of these molecules are complex, as infusion is the preferred and most usable route of administration for these biomolecules. Pharmacokinetic modulation and molecular stabilization are important aspects of nucleic acid-based research. The pharmacokinetic exposure and enhancement of these molecular forms are crucial goals. New technological advancement may be helpful to address these challenges and solve related issues [28,29,30,31,32,33]. Although there is huge scope for AI in drug delivery innovation and drug discovery, it still presents some major limitations that ultimately require human interference or intellectuals to interpret the complex results. The major contributions of AI predictions are based on the datasets, but the interpretation of the results, owing to the gray zone, require human interference to reach the appropriate conclusion. AI can experience issues with algorithm bias regarding the processing of information for predictions and the assessment of hypotheses. Moreover, it is not uncommon for docking simulations to result in the discovery of inactive molecules [34]. Therefore, a critical analysis of these parameters still requires human involvement for effective decision-making and cross-verifications, to rule out system bias issues. Nevertheless, there is huge potential in AI for possible application, and thus, extensive work may be able to reduce the limitations associated with AI and make it effective and reliable [35].

Regarding AI, the methodology employed involves the utilization of machine learning or its subsets, such as deep learning and natural language processing. The learning process can be either supervised or unsupervised, and the type of algorithm employed is also a crucial factor. Supervised learning is a machine learning methodology that involves the use of known inputs (features) and outputs (labels or targets), as opposed to unsupervised learning, which deals with unknown outputs. The supervised approach involves the prediction of output, such as labels or targets, based on multiple inputs or features. On the other hand, unsupervised classification aims to create groups that are homogeneous in terms of features [36].

**Figure 2:**
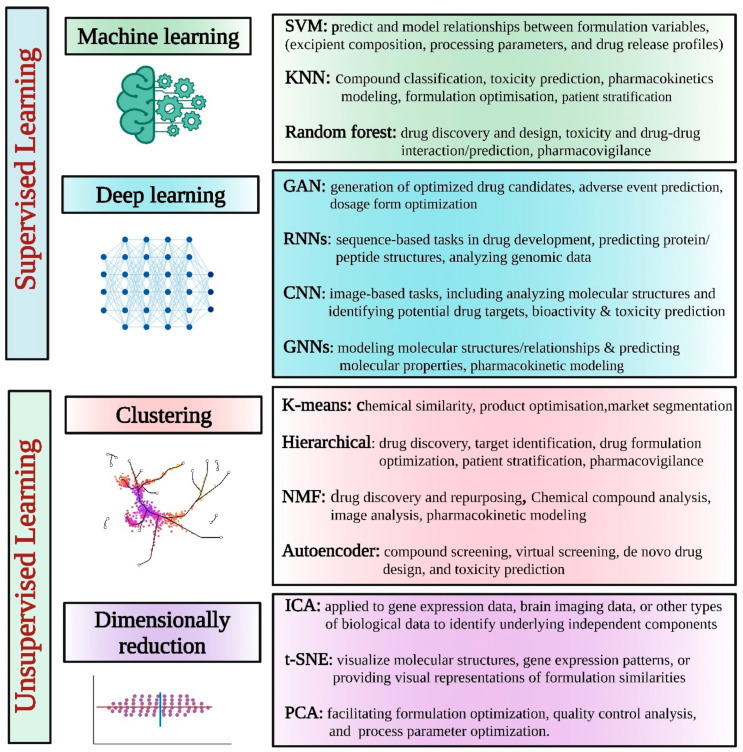
Different supervised and unsupervised AI learning models/tools for pharmaceutical applications.

In pharmaceutical product development, various AI models have been explored to enhance different aspects of the process. A list of commonly explored AI models in this domain is described in Table 1 and Figure 2.

### 2.1. Supervised AI Learning

Supervised learning refers to a type of machine learning in which an algorithm is trained on a labeled dataset, where the desired output is already known. The algorithm learns to map input data to the correct output by analyzing the patterns and relationships within the labeled data. This approach is commonly used in various applications, such as image recognition, natural language processing, and predictive modeling. Task-driven strategies involve setting specific goals for achieving desired outcomes from a given set of inputs. This approach utilizes labeled data to train algorithms for tasks such as data classification or outcome forecasting. The predominant supervised learning tasks are classification, which involves predicting a label, and regression, which involves predicting a quantity. A variety of techniques are available for solving supervised learning tasks, depending on the nature of the data in a given problem domain. These techniques include Naïve Bayes, K-nearest neighbors, support vector machines, ensemble learning, random forest, linear regression, support vector regression, and others [37]. It has several applications in the pharmaceutical industry, as described below:

- Drug Discovery and Design: Supervised learning algorithms can be used to predict the activity or properties of new drug candidates. By training on a dataset of known compounds and their associated activities, the model can learn patterns and relationships between molecular features and desired outcomes. This enables the prediction of the activity, potency, or toxicity of novel compounds, aiding in drug discovery and design [38].
- Predictive Maintenance and Quality Control: In pharmaceutical manufacturing, supervised learning can be utilized for predictive maintenance and quality control. By training on data from manufacturing processes, equipment sensor data, or quality testing results, the model can learn to predict equipment failure, product quality deviations, or process abnormalities, allowing for proactive maintenance and quality assurance [39].
- Drug Target Identification: Supervised learning techniques can help identify potential drug targets by analyzing biological data. By training on data that include information about genetic, proteomic, or transcriptomic features and their relationship to drug response or disease progression, the model can learn patterns and identify potential targets for further investigation [40].
- Disease Diagnosis and Prognosis: Supervised learning models can be used to diagnose diseases or predict patient outcomes based on medical data. By training on labeled datasets containing patient characteristics, clinical data, and disease outcomes, the model can learn to classify patients into different disease categories or predict disease progression or treatment response [41].
- Adverse Event Detection: Supervised learning algorithms can be applied to pharmacovigilance data to identify and classify adverse events associated with drugs. By training on labeled adverse event reports, the model can learn to recognize patterns and identify potential safety signals, helping in the detection and characterization of adverse events [42].
- Predictive Modeling for Clinical Trials: Supervised learning can be used to predict outcomes in clinical trials. By training on historical clinical trial data, including patient characteristics, treatment interventions, and trial outcomes, the model can learn to predict patient response, treatment efficacy, or safety outcomes. This information can guide trial design and optimize patient selection [43].

These are just a few examples of how supervised learning can be applied in the pharmaceutical industry. Supervised learning techniques, combined with appropriate feature selection, data preprocessing, and model evaluation, can provide valuable insights and support decision-making in various stages of pharmaceutical research, development, and manufacturing.

### 2.2. Unsupervised AI Learning

Unsupervised learning refers to a type of machine learning where the algorithm is not provided with labeled data. Instead, it is tasked with identifying patterns and relationships within the data on its own. This approach is often used in exploratory data analysis and can be useful for discovering hidden structures or clusters within a dataset. The approach being described is commonly known as a “data-driven methodology,” which aims to extract patterns, structures, or insights from unannotated data. There are several prevalent unsupervised tasks, including clustering, dimensionality reduction, visualization, finding association rules, and anomaly detection. Various unsupervised learning tasks can be addressed using popular techniques such as clustering algorithms (e.g., hierarchical clustering, K-means, K-medoids, single linkage, complete linkage, BOTS), association learning algorithms, and feature selection and extraction techniques (e.g., Pearson correlation, principal component analysis) based on the data’s characteristics [44,45]. Unsupervised learning techniques in AI can be valuable for pharmaceutical applications, particularly for exploratory analysis, pattern recognition, and data visualization, as described below:

- Clustering: Clustering algorithms group data points based on their similarities, allowing the identification of natural groupings or clusters within the data. In pharmaceutical applications, clustering can be applied to various datasets, such as gene expression profiles, chemical structures, or patient data, to uncover subgroups with similar characteristics. This can aid in target identification, patient stratification, and identifying distinct classes of compounds or diseases [46].
- Dimensionality Reduction: Dimensionality reduction techniques, such as principal component analysis (PCA) and t-distributed stochastic neighbor embedding (t-SNE), are used to reduce the complexity of high-dimensional datasets while preserving meaningful information. These methods can help visualize and explore complex datasets, identify key variables or features, and support decision-making processes. Dimensionality reduction can be applied to various types of pharmaceutical data, including gene expression data, drug activity profiles, or imaging data [47].
- Anomaly Detection: Anomaly detection algorithms identify rare or unusual data points that deviate significantly from the expected patterns. In the pharmaceutical industry, anomaly detection can be useful for detecting adverse events, identifying potential safety concerns, and uncovering data quality issues. Unsupervised anomaly detection techniques, such as the local outlier factor (LOF) or isolation forest, can help highlight abnormal patterns or data points that warrant further investigation [48].
- Association Rule Mining: Association rule mining techniques, such as the Apriori algorithm, aim to discover interesting relationships or associations between items in a dataset. In the pharmaceutical context, association rule mining can be applied to drug–drug interactions, adverse event data, or co-occurrence patterns between medical conditions and medications. These techniques can provide insights into potential drug interactions, identify medication patterns, or support pharmacovigilance activities [49].
- Topic Modeling: Topic modeling algorithms, such as latent Dirichlet allocation (LDA), extract latent topics or themes from large text datasets. In the pharmaceutical industry, topic modeling can be used to analyze the scientific literature, clinical trial reports, or social media data to identify key research themes, emerging trends, or patient sentiments. This can aid in literature mining, competitive intelligence, or understanding patient perspectives [50,51].

Unsupervised learning techniques offer valuable insights and exploratory analysis in pharmaceutical applications. However, it is important to note that the interpretation of results from unsupervised learning methods often requires domain expertise and further validation to extract actionable knowledge and ensure the reliability of the findings.

**Table 1:** Top 10 list of commonly used AI models in the pharmaceutical industry.

AI/Machine Learning Models	Description/Usage	References
Generative Adversarial Networks (GANs)	GANs are widely used in drug product development to generate novel chemical structures and optimize their properties. GANs consist of a generator network that creates new molecules and a discriminator network that evaluates their quality, leading to the generation of structurally diverse and functionally optimized drug candidates.	[52]
Recurrent Neural Networks (RNNs)	RNNs are commonly employed for sequence-based tasks in drug development, such as predicting protein structures, analyzing genomic data, and designing peptide sequences. They capture sequential dependencies and can generate new sequences based on learned patterns.	[53]
Convolutional Neural Networks (CNNs)	CNNs are effective in image-based tasks, including analyzing molecular structures and identifying potential drug targets. They can extract relevant features from molecular images and aid in drug design and target identification	[54]
Long Short-Term Memory Networks (LSTMs)	LSTMs are a type of RNN that excel in modeling and predicting temporal dependencies. They have been used in pharmacokinetics and pharmacodynamics studies to predict drug concentration-time profiles and evaluate drug efficacy.	[55]
Transformer Models	Transformer models, such as the popular BERT (Bidirectional Encoder Representations from Transformers), have been employed in natural language processing tasks in the pharmaceutical domain. They can extract useful information from the scientific literature, patent databases, and clinical trial data, enabling researchers to make informed decisions in drug development.	[56]
Reinforcement Learning (RL)	RL techniques have been applied to optimize drug dosing strategies and develop personalized treatment plans. RL algorithms learn from interactions with the environment to make sequential decisions, aiding in dose optimization, and improving patient outcomes.	[57]
Bayesian Models	Bayesian models, such as Bayesian networks and Gaussian processes, are employed for uncertainty quantification and decision-making in drug development. They enable researchers to make probabilistic predictions, assess risks, and optimize experimental designs.	[58,59]
Deep Q-Networks (DQNs)	DQNs, a combination of deep learning and reinforcement learning, have been used to optimize drug discovery processes by predicting the activity of compounds and suggesting high-potential candidates for further experimentation.	[60,61]
Autoencoders	Autoencoders are unsupervised learning models used for dimensionality reduction and feature extraction in drug development. They can capture essential characteristics of molecules and assist in compound screening and virtual screening.	[62,63]
Graph Neural Networks (GNNs)	GNNs are designed to process graph-structured data, making them suitable for drug discovery tasks that involve molecular structures. They can model molecular graphs, predict properties, and aid in virtual screening and de novo drug design.	[64,65]

## 3. AI for Drug Discovery

AI has revolutionized drug research and discovery in numerous ways. Some of the key contributions of AI in this domain include the following:

### 3.1. Target Identification

AI systems can analyze diverse data types, such as genetic, proteomic, and clinical data, to identify potential therapeutic targets. By uncovering disease-associated targets and molecular pathways, AI assists in the design of medications that can modulate biological processes.

### 3.2. Virtual Screening

AI enables the efficient screening of vast chemical libraries to identify drug candidates that have a high likelihood of binding to a specific target. By simulating chemical interactions and predicting binding affinities, AI helps researchers prioritize and select compounds for experimental testing, saving time and resources.

### 3.3. Structure-Activity Relationship (SAR) Modeling

AI models can establish links between the chemical structure of compounds and their biological activity. This allows researchers to optimize drug candidates by designing molecules with desirable features, such as high potency, selectivity, and favorable pharmacokinetic profiles.

### 3.4. De Novo Drug Design

Using reinforcement learning and generative models, AI algorithms can propose novel drug-like chemical structures. By learning from chemical libraries and experimental data, AI expands the chemical space and aids in the development of innovative drug candidates.

### 3.5. Optimization of Drug Candidates

AI algorithms can analyze and optimize drug candidates by considering various factors, including efficacy, safety, and pharmacokinetics. This helps researchers fine-tune therapeutic molecules to enhance their effectiveness while minimizing potential side effects.

### 3.6. Drug Repurposing

AI techniques can analyze large-scale biomedical data to identify existing drugs that may have therapeutic potential for different diseases. By repurposing approved drugs for new indications, AI accelerates the drug discovery process and reduces costs.

### 3.7. Toxicity Prediction

AI systems can predict drug toxicity by analyzing the chemical structure and characteristics of compounds. Machine learning algorithms trained on toxicology databases can anticipate harmful effects or identify hazardous structural properties. This helps researchers prioritize safer chemicals and mitigate potential adverse responses in clinical trials.

Overall, AI-driven approaches in drug research and development offer the potential to streamline and expedite the identification, optimization, and design of novel therapeutic candidates, ultimately leading to more efficient and effective medications [66].

For example, in silico target fishing technology (TF) is used in pharmaceuticals for biological target prediction based on chemical structure. This information is provided depending on the information available in the chemical database in the biological annotated form. Along with this, several other methods, such as data mining and docking of the chemical structure, were used for the exploration of the mechanism of action along with target class information required for effective planning. The target fishing technique was used in drug discovery with the help of machine learning along with cheminformatics tools. These two are used to obtain detailed knowledge related to the proper analysis of complex structures and the design of novel drug ingredients for the effective treatment of complex diseases. The routine drug discovery methods run by different industries are quite costly, as they involve several complicated events that must be addressed properly to conclude, such as the selection and identification of the target proteins and the mechanism of action of the small molecules in depth. To speed up this process, the TF was applied, which assisted in reducing the total experimental cost during the drug development processes. The reference molecules are used for the prediction of the ligand-target with the help of the 3D descriptors. This technique was used for the identification of the high binding ability of diethylstilbestrol, while the TF technique is widely implemented for the study of the phytopharmacology of the drug along with monthly similarity assessments. It is a computational and a proteomics-based method, which is based on the ranking of the data points depending on the similarity of data fusion along with drug targets. It is also used for the prediction of potential toxicities for the ligand-based approach used in drug discovery. Some of the critical points required in the drug development and drug discovery phases, such as novel target identification, selection, prediction of the phytopharmacological profiles, and prediction of the adverse effects associated with novel therapeutic indications, are explored with the TF. For these events, the bioactive compound similarity is applied for target identification to that of the unrecognized compounds. Some of the drugs that have been successfully characterized by using this method are loperamide and emetine, along with methadone, while the targets identified for the same are muscarinic, adrenergic, and neurokinin receptors [2,67,68,69,70,71,72,73,74,75].

The field of drug discovery has seen significant advancements with the use of AI models and tools. Some of the popular AI model tools used for drug discovery are described in Table 2. These are just a few examples of the AI model tools available for drug discovery. The field is rapidly evolving, and new tools and models are continuously being developed to accelerate the discovery of new drugs.

## 4. AI Tool Application in Dosage Form Designs

**Figure 3:**
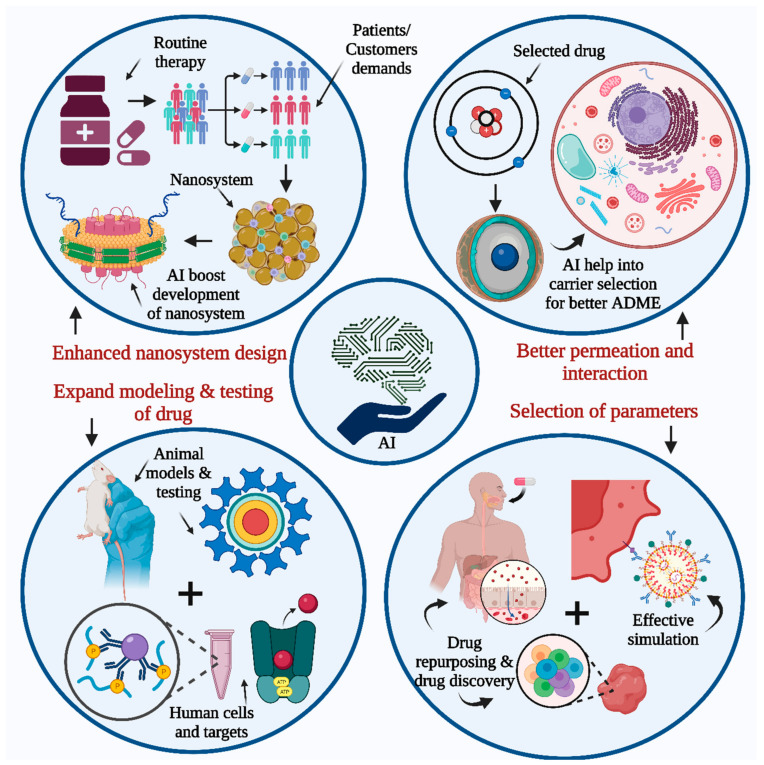
AI contribution to drug development and research. AI can be used to enhance nanosystem design, expand the present drug testing modeling system, and increase the accuracy of parameter and factor selection in drug design, drug discovery, and drug repurposing methods. It also helps to better understand the mechanism of membrane interaction with the modeled human environment by studying drug permeation, simulation, human cell targets, etc.

The human body system is divided into several compartments to understand the impact of drug delivery. The compartments are further simplified based on biological membranes. Physicochemical barriers are vital for biological compartments and can be implemented based on the mode of drug delivery inside the body. One of the most significant criteria for efficient drug delivery system monitoring is the rate of permeation based on the route of administration. The orally administered drug, after entering the gastric environment, must permeate through the intestinal or gastric epithelium. This step is vital for the further distribution of the drug into the bloodstream. The distribution step conveys the drug to the target site, which can be tissue or any of the specific cellular components [76,77,78,79,80]. Intracellular molecules can also act as targets for drug entry into the body. Most of the permeation of drugs is facilitated through biological barriers, either passively or actively. Passive diffusion is based on the drug’s molecular features. The in silico models are used to predict drug distribution through computation analysis, but these results are somewhat different from the actual drug distribution study. The drug’s interaction with biological components and the availability of the drug in biological environments have a great impact on the drug’s fate in the body. This process is governed by the molecular features of the drug. For many biologically active entities and small molecules, passive permeation is inefficient and requires a specific drug delivery system. The active permeation process is driven by membrane transport and depends on complex biological interactions. This complex process must be explored by using many specific parameters through computation and systematic modeling approaches. This newer computational model is used to study the pharmacokinetic parameters of the drug delivery system. One of the major loopholes present in the research and development of the pharmacy industry is the predictability of preclinical models. The predictability assumption is based on the selected parameters, and the same applies to complex in silico models as well. All these cases are linked to drug interactions with membranes and can be better analyzed by the modeled environment, as presented in Figure 3. This modeled environment can be studied and analyzed more effectively through AI [81,82,83]. AI provides sophisticated technology for the analysis of such multilayer data. The thoroughness of the analysis will contribute to a better understanding of the research units. The systematically applied model along with parameter evaluation is based on many factors, such as simulation, scoring, and refinement, in each step of the research to determine the best outcomes. AI could provide an automated system that can be implemented for all these functions for better guessing and predicted refinement of the data for consistent improvement. For better AI training in the biological environment, a proper understanding of the drug–biological interaction is essential, which is indicated by the system biology type of the databases. Pharmacokinetic studies can be performed using many novel AI technologies, such as artificial neural networks. Along with this, many databases are provided by AI, such as chemical, genomic, and phenotypical databases, for a better understanding of the drug interaction and the effective study of the molecules’ complex unit roles within the same. Some of the methods are also applied to study the impact of the drug delivery system on the pharmacokinetics of the drug, for an effective understanding of the disposition and toxicity. Many new approaches to drug delivery systems involve the design of quality attributes along with critical attributes and studying their impacts on experimental trials before actual experiments.

The benefits of AI are that it collects information from multiple sources and provides indications for the selected drug delivery system to work as per the anticipated results. The evaluation of the molecular information, patient data, and pharmacokinetic data are considered part of the complex data for analysis for the possible selection of the best active pharmaceutical against patient diseases or requirements. The passive type of AI is implemented for the identification of molecular entity features against those of known molecules for comparison. Effective treatment depends on the accuracy of the selection of drug delivery systems, which are provided by AI.

**Figure 4:**
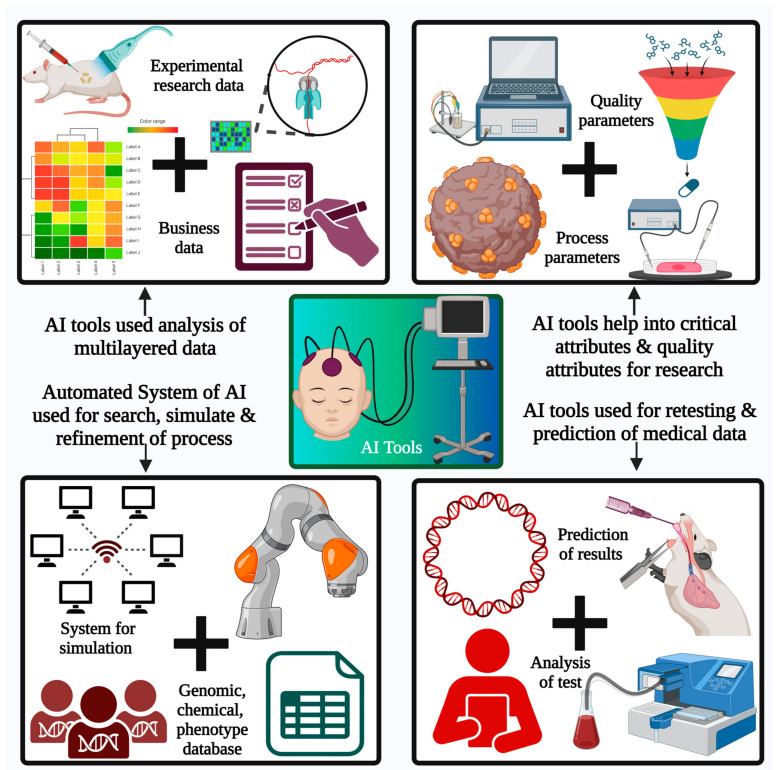
Application of AI tools in the pharma sector. AI tools are helpful for the analysis of multilayered data. Automated AI systems are used to perform effective searches, simulations, and refinements of data and processes involved in research and product development. The system biology database, chemical database, genomic database, phenotypic database, and AI bots are used for better exploration of drug models, drug release, and activity predictions along with recommendations for effective drug delivery systems.

AI is also useful for the drug discovery process along with the drug repurposing method. This addresses the application of the existing therapeutics to that of the new disease. The requirement of the patients and disease condition are major factors contributing to formulation, pharmacokinetics, and drug development. One of the major challenges associated with the application of AI in full scope to develop delivery systems is the availability of databases with detailed information. This is required for the evaluation of the models, along with parameters, in an unbiased way. AI provides help for future applications by using current knowledge. A large quantity of the data can be handled or digested by using AI tools for a better approach to the rational design of the product, as presented in Figure 4. A more vigorous codification inside the knowledge database can be performed with excellent self-supervised experimental results and related to proper parameter recording [84,85,86,87,88,89,90].

## 5. AI for Drug Delivery

The integration of AI and big data in the field of pharmaceutics has led to the development of computational pharmaceutics, which aims to enhance drug delivery processes by utilizing multiscale modeling approaches. Computational pharmaceutics employs AI algorithms and machine learning techniques to analyze large datasets and predict drug behavior (Table 3). By simulating drug formulation and delivery processes, researchers can evaluate various scenarios and optimize drug delivery systems without the need for extensive trial-and-error experiments. This accelerates the drug development timeline, reduces costs, and increases productivity. Computational pharmaceutics involves modeling drug delivery systems at different scales, ranging from molecular interactions to macroscopic behavior. AI algorithms can analyze complex relationships between drug properties, formulation components, and physiological factors to predict drug behavior at each scale. This allows for a more comprehensive understanding of drug delivery mechanisms and aids in designing efficient drug delivery systems. It helps in the prediction of the physicochemical properties of the drug, the in vitro drug release profile, and the stability of the drug. The same technology is also implemented for the better assessment of in vivo pharmacokinetic parameters and drug distribution along with in vivo-in vitro correlation studies. By utilizing the right set of AI tools, researchers can identify potential risks and challenges associated with drug delivery systems early in the development process. This allows for proactive modifications and adjustments to mitigate risks and optimize drug performance. The use of AI and computational modeling reduces the reliance on time-consuming and expensive trial-and-error experiments, minimizing the chances of unforeseen outcomes [91,92].

### 5.1. AI for Oral Solid Dosage Form Development

AI involves the use of advanced tools and software to achieve human-like capabilities. Such innovation has helped in many sectors, such as the pharmaceutical industry, especially in the product development phase over the past few years. The implementation of these technological innovations can save time, money, and resources required for manufacturing and proper distribution to end customers through the supply chain. It also provides a better platform to understand the impact of process parameters on the formulation and manufacturing of products.

Run Han et al. explored the utilization of machine learning methods for the prediction of solid dispersion stability for six months. Hanlu Gao et al. investigated the application of machine leaching for solid dispersion dissolution studies. They used a random forest algorithm to generate a classification model that further helps to distinguish between the spring and parachute types of dissolution profiles. It also contributed to maintaining supersaturation with eighty-five percent accuracy and eighty-six percent sensitivity. The time-dependent drug release was predicted based on the regression model created by the random forest algorithm [93].

In the pharmaceutical market, solid dosage forms are predominant, and tablets are one of the dominant dosage forms in this domain. The preparation of the tablet includes many factors based on the type of tablet. AI can help in the search for optimized formulation and the study of the desired attributes involved in the same. AI is also expected to process obligations with the help of automated algorithms and technologies. The implementation of AI also poses a challenge to the regulatory authorities to redefine the policies regarding current good manufacturing practice (cGMP). Different technologies in AI, such as artificial neural networks (ANNs), fuzzy logics, and neural networks, along with genetic algorithms, are implemented for the development of solid dosage forms and a better understanding between the inputs and outputs for processing and operations. ANN is used for better prediction abilities for solid dosage forms, while genetic algorithms are used to predict the results obtained from the utilization of input parameters [94].

**Table 3:** List of commonly explored AI models in pharmaceutical product development.

AI/Machine Learning Models	Description/Usage	References
Genetic Algorithms	Genetic algorithms are optimization techniques inspired by the principles of natural selection and genetics. They can be applied to optimize formulation compositions, drug release profiles, and process parameters to achieve desired dosage form characteristics.	[95]
Artificial Neural Networks (ANNs)	ANNs have been employed to model and optimize drug release kinetics from different dosage forms. They can assist in determining optimal formulations and predict the release behavior of active pharmaceutical ingredients (APIs) under various conditions.	[96]
Support Vector Machines (SVMs)	SVMs have been used in dosage form optimization to predict and model relationships between formulation variables, such as excipient composition, processing parameters, and drug release profiles. They aid in optimizing formulation design space.	[97]
Particle Swarm Optimization (PSO)	PSO is a population-based optimization algorithm that can be used for dosage form optimization. It has been applied to optimize particle size distribution, dissolution profiles, and other formulation parameters.	[98]
Artificial Intelligence-based Expert Systems	Expert systems utilize AI techniques, including rule-based systems and fuzzy logic, to simulate the decision-making process of human experts. They can be applied to dosage form optimization by considering multiple formulation and process variables.	[99]
Monte Carlo Simulation	Monte Carlo simulation methods have been used to optimize drug product performance by considering uncertainties and variability in formulation and process parameters. They aid in robust formulation and process design.	[100]
Computational Fluid Dynamics (CFD)	CFD simulations enable the optimization of fluid flow and mixing within dosage form manufacturing processes, such as granulation, coating, and drying. They help in designing efficient and uniform processes.	[101,102]
Response Surface Methodology (RSM)	RSM is a statistical technique that helps optimize dosage form formulations by modeling and analyzing the relationship between multiple variables and their effect on formulation responses. It aids in understanding and optimizing formulation parameters.	[103,104,105]
Artificial Neural Network–Genetic Algorithm (ANN-GA) Hybrid Models	Hybrid models combining ANN and GA techniques have been used for dosage form optimization. They can efficiently search the formulation space to identify optimal solutions and predict formulation characteristics.	
Multivariate Analysis Techniques	Multivariate analysis methods, such as principal component analysis (PCA) and partial least squares (PLS), have been employed in dosage form optimization. They aid in identifying critical formulation variables, reducing dimensionality, and optimizing formulation performance.	[106,107,108]

Tablets are a highly used solid dosage, occupying a substantial portion of the market within the drug delivery segment. The process of creating this product involves the utilization of active pharmaceutical ingredients along with excipients, which are subsequently compressed or molded to achieve the intended form and dimensions. Numerous excipients are incorporated into tablets to manage the desired product outcome, including tablet disintegration, dissolution, and drug release. These factors are predetermined by the formulator to meet the specific needs of the target patient population. Certain excipients are essential in facilitating the manufacturing process, including glidants and lubricants. AI can also be utilized in the context of systemic drug delivery to predict drug release. Additionally, it is employed to investigate the effects of crucial processing parameters that are integral to tablet manufacturing, with the potential to ensure consistent quality control measures. Certain AI applications have been utilized to identify defects in tablets [109,110].

#### 5.1.1. Prediction of Dug Release through Formulations

The prediction of drug release certainly has the potential for stable quality control. Drug release studies are performed through in vivo and in vitro methods, which are treated as fundamental technologies regularly evaluated or tested during product development. The release of the drug from oral solid dosage forms is based on the contribution of critical material attributes along with the processing parameters. Some of the common factors affecting drug release include compaction parameters such as the pressure used for tablet hardness setting, geometric aspects of the tablets, and drug loading characteristics. Many analysis techniques, including spectrophotometric analysis methods, have been implemented, or drug release studies are usually required for extensive analysis.

**Figure 5:**
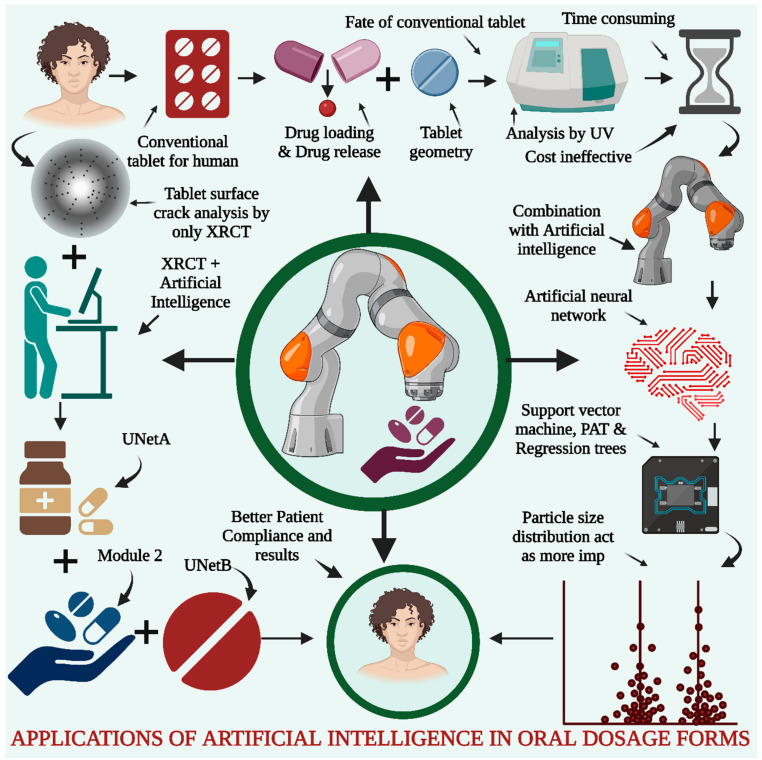
AI for Oral Dosage Forms. Conventional tablet analysis is performed by screening many factors, such as drug release, drug loading, and study of the tablet geometry and hardness, by using in-process quality control tests along with ultraviolet spectrophotometry. These methods are often time-consuming and cost-ineffective to the industry. To address these issues, the combination of such traditional techniques along with AI was performed by using ANN, SVM, PAT, and regression trees. The data analysis and drug release predictions indicated that particle size distribution was a crucial factor for the same. Defective tablet surface crack analysis is performed by XRCT in combination with AI, containing three modules for distinguishing features for effective application in the healthcare sector.

The drug release results must be set as per the formulator’s requirements and require repetitive testing and preparation of the batches to obtain an optimized batch, which makes this task tedious and time-consuming [111]. AI is implemented in the drug formulation and will assist in the prediction of drug release; hence, there is a limited number of runs required to optimize the batch, which further induces a reduction in the work and cost during pilot batch scale and production processes. AI can help predict the drug release profiles and dissolution profiles and explore the disintegration time for the effective selection of the best batch for further scale processing. Some researchers have implemented AI algorithms for the prediction of dissolution profiles into the hydrophilic matrix type of sustained-release tablets with the help of artificial neural networks (ANNs). The support machine vector (SVM), as well as regression analysis, are also implemented during the analysis of the data and prediction of the dissolution profile. The data for the modeling study of drug release were obtained with the help of process analytical technology (PAT) along with critical material attributes. The particle size distribution was found to be the most crucial variable during model prediction. Finally, the ANN was implemented for the identification of the most accurate models as part of the evaluation metrics, as presented in Figure 5 [112,113].

#### 5.1.2. Application of AI for 3D-Printed Dosage Forms

The application of AI in the field of 3D-printed dosage forms has revolutionized pharmaceutical manufacturing by enabling personalized medicine and enhancing drug delivery systems. AI algorithms can optimize the design and formulation of 3D-printed dosage forms based on patient-specific factors, such as age, weight, and medical history, leading to tailored drug therapies. By leveraging machine learning and computational modeling, AI can analyze large datasets and simulate the behavior of 3D-printed dosage forms, allowing for the rapid prototyping and optimization of drug release profiles, dosage strengths, and geometries. AI also aids in predicting and overcoming potential manufacturing challenges, optimizing printing parameters, and ensuring quality control. Furthermore, AI-driven feedback systems can continuously improve the 3D-printing process by learning from real-time data, enhancing accuracy, reproducibility, and scalability. Overall, the application of AI in 3D-printed dosage forms holds tremendous potential in advancing personalized medicine and improving patient outcomes [114,115].

The 3D-printed tablets are prepared by using the fused-filament type of fabrication, jetting of the binder, utilization of laser sintering, and pressure microsyringe. Some of the crucial processing parameters impacting the 3D-printed tablets are the temperature of the nozzle and platform along with the speed of the printing. Obeid et al. demonstrated the impact of the processing parameters on a 3D-printed tablet containing diazepam and its subsequent drug release study with the help of an ANN model. They explored the infill pattern, infill density, and other input variables for effective drug dissolution into 3D-printed tablets. The interactions between the different variables were evaluated with the help of self-organizing maps. Further modeling studies were performed by keeping the infill density along the surface area and volume ratio as the crucial factors contributing to the same. The higher dissolution resulted after extensive testing and ANN modeling along with validation [116,117].

#### 5.1.3. Application of AI for the Detection of Tablet Defects

The application of AI in the detection of tablet defects has revolutionized quality control processes in pharmaceutical manufacturing. AI algorithms and computer vision techniques are employed to analyze images of tablets, enabling the automated and efficient detection of defects such as cracks, chips, discoloration, or variations in shape and size. By training AI models on large datasets of labeled images, the system learns to accurately classify and identify different types of defects, achieving high levels of precision and recall. Conventional methods, such as X-ray computed tomography, have been used to analyze the internal structure of tablets, but they are still time-consuming and affect the demand for the rapid production of tablets. Deep learning is implemented along with X-ray tomography to detect tablet defects. Ma et al. explored the application of neural networks for tablet defect detection with the help of image analysis completed through X-ray tomography. These researchers have manufactured several batches of tablets by using excipients such as microcrystalline cellulose along with mannitol. The prepared batches were analyzed with the help of the so-called image augmentation strategy. Three different models were used during the same research, including UNetA, which is applicable for the identification of distinguished characteristics of tablets from those of bottles. Module 2 was used for the identification of individual tablets with the help of augmented analysis. The internal cracks in the internal structure of the tablet were analyzed with the help of UNetB. Such UNet networks have been used to check tablet defects with better accuracy and thus provide ease of identification of defects with significant reductions in time, financial costs, and workload [118,119]. This AI-powered detection not only improves the speed and accuracy of defect identification but also reduces the dependence on manual inspection, minimizing human errors and subjective judgment. The real-time monitoring capabilities of AI systems ensure the prompt detection of defects, facilitating timely intervention and preventing the release of faulty tablets into the market. Ultimately, the integration of AI into tablet defect detection enhances product quality, increases productivity, and ensures the safety and efficacy of pharmaceutical products.

#### 5.1.4. AI for the Prediction of Physicochemical Stability

AI has emerged as a powerful tool for predicting the physicochemical stability of oral dosage forms in pharmaceutical research. By leveraging machine learning algorithms and computational models, AI can analyze and interpret large datasets, including drug properties, formulation parameters, and environmental conditions, to predict the stability of oral formulations. AI models can assess factors such as drug degradation, interaction with excipients, and environmental effects on formulation stability. These predictive capabilities enable researchers to optimize formulation designs, identify potential stability issues early in the development process, and make informed decisions to enhance the shelf life and efficacy of oral dosage forms. The integration of AI into stability prediction contributes to more efficient and cost-effective drug development processes, ultimately leading to the delivery of safe and effective medications to patients. Some researchers have studied the utilization of machine learning for the determination of solid dispersion with the help of several algorithms. Han et al. explored the application of machine learning for the prediction of solid dispersion by implementing ANN along with K-nearest neighbor (KNN) algorithms as well as a light gradient boosting machine (LightGBM). The SVM was also applied in the same way. KNN is a nonparametric type of supervised learning classifier. It was used to classify or complete the predictions for the grouping along with the individual data point [120]. The free- along with the open-source distributed gradient boosting framework implemented with machine learning was the LightGBM. It is usually utilized for ranking assessments and classification along with machine learning tasks. In this study, approximately fifty drug molecules with six hundred forty-six data points for physical stability were collected from the public database and implemented for the training model. The generation of the database was performed with the help of molecular representations and molecular descriptors, such as molecular weight, along with the hydrogen bond acceptor count. The melting point and heavy atom count also acted as molecular descriptors. For three months, an accelerated stability study was conducted for the further evaluation of the model performance as a part of the physical stability prediction. They found an overall 82% accuracy for the same experiments [121,122].

#### 5.1.5. Contribution of AI to Dissolution Rate Predictions

The dissolution rate of a drug, which refers to the rate at which it dissolves in a biological fluid, is a crucial parameter that determines its bioavailability and therapeutic effectiveness. AI has made significant contributions to the prediction of dissolution rates, aiding in the optimization of drug formulations and dosage forms. Through the analysis of vast amounts of experimental data, AI models can identify key physicochemical properties and molecular features that influence the dissolution process. These models leverage machine learning algorithms to learn complex patterns and relationships between drug properties and dissolution rates, enabling accurate predictions. By providing insights into the dissolution behavior of different drug formulations, AI facilitates the design of more effective drug delivery systems and helps in the selection of optimal formulation strategies for enhanced drug solubility and absorption. This advancement in dissolution rate prediction powered by AI empowers pharmaceutical scientists with valuable tools to accelerate drug development, optimize formulation strategies, and ultimately improve patient outcomes [97].

Many researchers have studied the dissolution profiles of routine drugs, and they have documented the rapid dissolution of some drugs and supersaturation of related drugs. Amorphous drug recrystallization and precipitation are also crucial factors associated with this process. Some studies have shown that solid dispersions do not precipitate due to the addition of excipients. Dong et al. explored a method for predicting dissolution along with the dissolution rate by using AI for at least 50 active pharmaceutical ingredients along with 25 polymers. Some of the AI algorithms they have used include SVM, LightGBM, and extreme grading boosting (XGBoost) [123]. XGBoost is a scalable machine learning-related library consisting of a distributed gradient-boosted decision tree, which is helpful in the prediction of problems associated with unstructured data, including images and texts. The artificial neural network was used to outperform all other types of algorithms or frameworks. In the same study, the same team used molecular computational software for the descriptors for the active pharmaceutical ingredients as well as the polymers. The input variables selected for the same study were temperature, drug loading, and volume, while dissolution was recognized as that of the binary output including precipitation or supersaturation. The dissolution rate was considered the research output for the same and resulted in the greater accuracy of the prediction of the results for the dissolution profiles of the selected active pharmaceutical ingredients, along with the polymers [124,125].

### 5.2. AI for Nanomedicine

By harnessing AI’s capabilities in data analysis, pattern recognition, and optimization, nanomedicine researchers can accelerate the development of novel nanoscale interventions, improve diagnostics, enhance drug delivery, and advance personalized medicine. AI in nanomedicine holds great potential for revolutionizing healthcare by enabling precise and targeted therapeutic approaches at the nanoscale [126]. Nanoparticles are used for targeted drug delivery, imaging, and sensing. AI algorithms can aid in designing and optimizing nanoparticles by predicting their physicochemical properties, stability, and efficacy. This helps researchers develop nanoparticles with desired characteristics for specific applications. Nanomedicines are used effectively as drug delivery carriers for drugs or combinations of drugs based on the concept of drug synergy, especially for the treatment of cancer patients. They contain major impactful inputs, such as drug selection, dose selection, and stimuli-responsive material selection. The deep learning type of algorithm was used for melanoma and has shown great accuracy in caring for patients and assisting in diagnostic procedures [127,128].

AI algorithms can model the behavior and interactions of nanoscale materials within biological systems. This enables the prediction of nanoparticle behavior, drug release kinetics, and potential toxicity, facilitating the development of safe and effective nanomedicine formulations. AI can be used in nanosensors and biosensors for the real-time monitoring of biomarkers, drug levels, or disease progression. These sensors can provide continuous feedback to healthcare providers, enabling timely interventions and personalized treatment adjustments [129].

The AI-based database is useful for scaling up nanocarriers by using an automated system. AI is also used in nanocarrier drug delivery systems, particularly in the optimization of nanocarriers and drug compatibility testing by using computational approaches. Such approaches are used for the evaluation of drug loading, formulation stability, and drug retention. Thus, AI intervention contributes to the enhancement of the therapeutic nanocarriers required for specific cell types for the treatment of tumors. Yuan He et al. studied the application of machine learning methods to the prediction of nanocrystals prepared by high-pressure homogenization along with the wet ball milling method. The demands for a repetition of the experiments can also be decreased by using computational techniques through Monte Carlo simulations and molecular dynamics, along with theoretical techniques. The simulation techniques are helpful for quantitative measurements in critical experiments. AI is also implemented for the creation of the database repository required for nanocarriers, which further helps in the determination of 3D structures along with physical and chemical property investigations in collaboration with structural nanobiology. Such repositories are essential to investigate the relationship between nanocarrier structure and toxicological, physical, and biological data [130,131,132,133,134,135,136,137]. In another study performed by Lutz Nuhn for the application of AI for better analysis, it was found that AI helped to reveal the heterogeneous vascular permeability for prepared nanoparticle-based drug delivery systems using an analysis of single blood vessels. Such findings may help in the design of a protein nanoparticle drug delivery system to obtain an active type of transendothelial permeability into tumors [138]. Zhoumeng Lin et al. used AI for better assessment with a PBPK modeling approach to study cancer medicine effectively. The same is also helpful to obtain a better understanding of the causes of low nanoparticle tumor delivery efficacy [139].

### 5.3. AI Application for Parenteral, Transdermal and Mucosal Route Products

Injectables, biologics, and other complicated formulations can be developed and manufactured using AI. Predicting complicated drug formulation physicochemical parameters using AI systems may help formulation development. AI models optimize pH, solubility, stability, and viscosity by analyzing formulation components, excipients, and manufacturing processes. This helps create stable parenteral formulations. AI can optimize parenteral product production for quality, efficiency, and variability. AI algorithms may discover process factors that affect product qualities and offer appropriate modifications by analyzing real-time process data. Thus, product consistency, batch failures, and manufacturing productivity increase. AI algorithms may find trends and product quality variations in huge datasets from analytical tests, including particle size analysis, spectroscopy, and chromatography. This helps identify and fix quality concerns early, assuring high-quality goods. AI models may anticipate contamination, stability, and regulatory deviations using historical data and process factors. AI-based monitoring systems may analyze important process parameters in real time during parenteral product manufacture. AI algorithms can identify abnormalities and forecast deviations and take quick action by combining data from sensors, instruments, and process controls. This maintains product quality and minimizes noncompliance. AI optimizes maintenance procedures for complicated parenteral product manufacturing equipment. AI models analyze sensor data, equipment performance history, and maintenance records to forecast equipment failure or deterioration and schedule proactive maintenance. This saves unnecessary downtime, boosts output, and cuts maintenance. AI can help ensure parenteral and complex biological product regulatory compliance. AI algorithms may analyze compliance, detect possible noncompliance concerns, and provide process improvement ideas by analyzing process data and product properties. This aids GMP compliance and regulatory compliance [140].

For example, AI was used in the inspection of the particles to check whether the particles were swimming, sinking, or sticking into the inner side of the container. For proper inspection of the individual particles, the optical setup, strategy, algorithm, and inspection were recommended. The particle tracking algorithm along with image subtraction was used for the analysis of the floating particles. The liquid inside the container is allowed to move so that the behavior of the moving particles can be recorded with the help of high-resolution images, while the particle movement direction can also be traced with the help of AI. The deep learning algorithm is also used for the proper isolation of the particles. One of the greater issues associated with parenteral batch flaws is bubble formation, which is normally not harmful to patients, but there is a great need to distinguish between particles and bubbles. The AI-based image processing type of algorithm was used for these types of visual inspection and the issues associated with them. One of the other camera-based applications of AI was surface crack detection by using surface qualifies 7500, which is used to analyze hundreds of millions of data points per second with the help of graphical processing subunits [127,128,140,141,142]. Manufacturers may optimize product performance, decrease manufacturing hazards, and provide safe and effective parenteral and technologically advanced pharmaceutical products using AI data analysis, pattern recognition, and predictive modeling.

Bannigan et al. highlight the availability and potential of cutting-edge machine learning (ML) technologies in the field of pharmaceutical and materials science. They demonstrate that ML can accelerate the development of innovative drug delivery technologies by accurately predicting in vitro drug release from long-acting injectables (LAIs). The study emphasizes the interpretability of ML models, which can provide insights into the decision-making process. Although neural network models did not perform well due to the small dataset, tree-based models such as LGBM showed promise in reducing the time and cost associated with LAI formulation development. The study presents a proof-of-concept for ML in drug formulation and hopes to inspire more advanced and tailored ML approaches in the future [143,144].

The conventional trial-and-error approach in formulating ocular, transdermal, pulmonary and other mucosal drug delivery systems lacks in-depth understanding, making it inefficient for complex formulations. However, recent advancements in computational pharmaceutics, specifically machine learning and multiscale simulations, have opened up new possibilities. Recent progress in using molecular simulations, mathematical modeling, and PK/PD modeling for these drug delivery routes has led to more efficient product development. In silico modeling and simulations offer unique advantages by providing detailed insights and facilitating rational formulation design. The integration of in silico methodologies, overcoming data challenges, and interdisciplinary collaborations can lead to more efficient and objective-oriented drug formulation design in the era of Pharma 4.0 [145,146,147,148].

### 5.4. AI Tools for Biologics Product Development

AI helps create newer proteins, peptides, nucleic acid biologics and immunotherapeutics [144,145,146,147,148,149,150,151,152]. AI algorithms could help to build proteins and peptides with desired features [153,154,155,156,157]. AI models may produce therapeutic sequences with better stability, binding affinity, or immunogenicity by analyzing massive volumes of protein structure and function data. This allows for customized biologics with improved effectiveness and safety [158,159].

**Figure 6:**
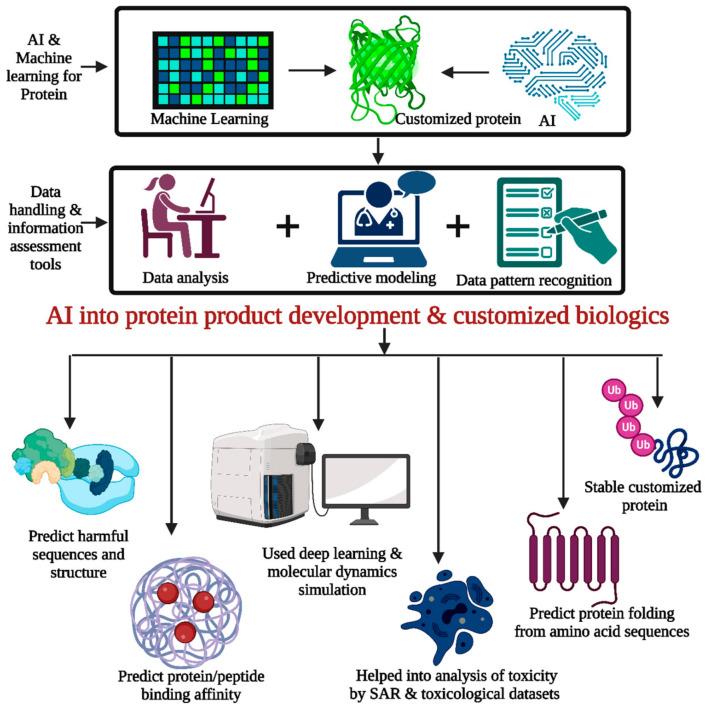
AI can contribute to protein development and customized biologics by using data analysis, predictive modeling, and pattern recognition tools for better improvisation in the protein development process and customized proteins. Knowledge of altered biological pathways and finding disease targets are required for the same. The prediction of protein folding from amino acid sequences and the use of deep learning and molecular dynamic simulation for better understanding can be performed by AI. The prediction of protein/peptide binding affinity and toxicity studies can be performed effectively by AI with the help of SAR and toxicological datasets.

AI systems can find therapeutic targets using genetic, proteomic, and clinical data. AI helps researchers build protein and peptide biologics that alter biological pathways or target illness-causing proteins by finding disease targets. AI models can predict protein folding from amino acid sequences. Understanding protein function and creating optimized biologics requires protein folding. Deep learning and molecular dynamics simulations can anticipate protein folding patterns, helping design stable and functioning biologics (Figure 6) [160].

AI algorithms predict protein/peptide-target molecule binding affinity. AI models may reliably estimate binding strength by training on huge protein–protein or protein–peptide datasets. This improves treatment effectiveness by choosing or creating biologics with a high affinity and specificity for targets. AI could help to optimize protein and peptide biologics formulations. Stability, aggregation tendency, and formulation factors affect biologic quality and effectiveness. AI algorithms can optimize formulation conditions and biologic stability and shelf life by analyzing protein physicochemical parameters, formulation components, and manufacturing processes [161].

AI algorithms predict protein and peptide biologic toxicity. AI systems can anticipate biologic adverse effects and immunogenicity by analyzing structure–activity relationships while being trained on toxicological datasets. This allows researchers to find and alter harmful sequences or structures. AI is being utilized to optimize clinical trials for protein and peptide biologics. AI algorithms are capable of predicting patient responses and refining trial procedures using patient data, illness features, and treatment results. This streamlines patient enrollment, study design, and personalized treatment [162,163,164]. AI has the potential to significantly enhance research, diagnostics, and therapeutics in the fields of exosomes, CAR T-cell therapy, and CRISPR/Cas9 [164,165,166].

By utilizing AI’s capabilities in data analysis, pattern recognition, and predictive modeling, the development of protein/peptide and gene therapy biologics can be accelerated, and the design and optimization of therapeutic molecules can be more efficient and targeted. AI holds immense potential to revolutionize the field by enabling the creation of novel biologics with enhanced properties and improving the success rate of biologic development [167].

### 5.5. AI in Medical Devices

The medical device is a sort of apparatus, implement, instrument, implant, or machine appliance as well as a reagent for specific medical purposes and can be used alone or in combination with the help of software or other related systems in vitro to address medical issues of patients. AI has made significant advancements in the field of medical devices, revolutionizing healthcare in various ways. Due to the pandemic, personalized medicine along with remote health monitoring has become essential and quite popular in many countries, which has boosted AI and machine learning applications in the healthcare sector. Some examples of how AI is being utilized in medical devices are described below:

- Diagnostic Assistance: AI algorithms can analyze medical imaging data such as X-rays, CT scans, and MRIs to assist healthcare professionals in detecting and diagnosing diseases. For example, AI-powered algorithms can help identify cancerous lesions in medical images or detect abnormalities in electrocardiograms (ECGs) [168].
- Remote Monitoring: AI-enabled medical devices can remotely monitor patients’ health conditions, allowing for the continuous tracking of vital signs and other relevant parameters. This is particularly useful for patients with chronic conditions who can receive personalized care from the comfort of their homes. AI algorithms can analyze the collected data and provide alerts or insights to healthcare providers [169].
- Wearable Devices: AI is integrated into wearable devices such as smartwatches, fitness trackers, and biosensors. These devices can monitor various health parameters, such as heart rate, sleep patterns, physical activity, and even blood glucose levels. AI algorithms help interpret the data and provide users with actionable insights for improving their health and well-being [170].
- Prosthetics and Rehabilitation: AI is used in advanced prosthetic devices to provide more natural movement and functionality. Machine learning algorithms can learn from user movements and adapt the prosthetic to better match the user’s intentions. AI can also assist in rehabilitation by analyzing motion and providing feedback to patients to improve their movements and track progress [171].
- Surgical Assistance: AI has found applications in surgical devices, aiding surgeons during procedures. For instance, robotic surgical systems use AI algorithms to assist surgeons in performing precise and minimally invasive procedures. AI can also analyze preoperative and intraoperative data to provide real-time guidance and improve surgical outcomes [172].
- Medication Management: AI-powered devices can help patients manage their medications effectively. Smart pill dispensers can remind patients to take their medications on time, dispense the correct dosage, and track adherence. AI algorithms can also analyze patient data, such as medical history and medication usage, to provide personalized recommendations for medication management [173].

These examples demonstrate how AI is integrated into medical devices to enhance diagnostics, monitoring, treatment, and patient care. AI’s ability to analyze large amounts of data, identify patterns, and provide personalized insights contributes to more accurate diagnoses, improved treatment outcomes, and better overall healthcare delivery. It also contributes to the development of new products for patient benefits and to effectively reaching out to new customer segments to captivate large businesses and create more business potential in the healthcare sector. Currently, medical technology-based companies are using AI in major sectors, such as diagnosis, prevention, and care, along with personalized medicine work for patients.

For example, Medtronic, a global medical technology company, has indeed developed innovative applications of AI to help patients with diabetes manage their condition effectively. One notable example is the Medtronic Guardian Connect system, which combines AI and continuous glucose monitoring (CGM) technology to provide real-time insights and support to individuals living with diabetes. In 2016, Medtronic collaborated with IBM Watson to develop the Medtronic Sugar IQ app, which serves as a mobile personal assistant for individuals managing diabetes. This app incorporates AI technology to provide valuable features for effective diabetes disease management. One of the major features of the Sugar IQ app is “insights.” The app analyzes the user’s glucose patterns over time, identifies trends, and provides personalized messages and notifications to the patient. These insights help individuals understand how specific actions, habits, and external factors impact their glucose levels. By gaining this understanding, users can make informed decisions and take proactive steps to manage their diabetes more effectively. The second important feature of the Sugar IQ app is “glycemic assistance.” The app utilizes AI algorithms to provide real-time guidance and recommendations to users based on their current glucose readings. If the glucose levels are trending high or low, the app can suggest actions to help the user maintain a more stable glucose range. This feature acts as a virtual assistant, providing personalized support and reminders to help users make appropriate choices regarding their diabetes management. Last, the Sugar IQ app incorporates a “food logging” functionality. Users can log their meals and track carbohydrate intake through the app. The app can then analyze the impact of different foods on glucose levels and provide insights into how specific meals or food choices affect blood sugar. This information enables individuals to make more informed dietary decisions, leading to better glycemic control. By combining AI technology with glucose monitoring and personalized messaging, the Medtronic Sugar IQ app offers valuable tools for individuals with diabetes. It helps users gain insights into their glucose patterns, provides real-time assistance in managing blood sugar levels, and assists in making informed decisions about diet and lifestyle choices. These features contribute to improving disease management and supporting patients in achieving better control of their diabetes [174,175,176,177,178].

## 6. AI for Pharmacokinetics and Pharmacodynamics

Drug development is a complex process that involves several stages, including drug discovery, preclinical studies, clinical trials, and regulatory approval. Pharmacokinetics and pharmacodynamics are crucial aspects of drug development, as they determine the optimal dosage, administration route, and safety of a drug in the body [85]. Traditional experimental methods for pharmacokinetics and pharmacodynamics studies can be time-consuming and expensive and may not always provide accurate predictions of drug efficacy and safety [179,180].

Traditionally, pharmacokinetics and pharmacodynamics studies have been conducted using experimental methods such as animal studies and human clinical trials. These methods have critical challenges, such as ethical concerns, sample size, and interindividual variability. Furthermore, these studies may not always provide accurate predictions of drug pharmacokinetics and pharmacodynamics in humans. To overcome these limitations, computational models and AI methods have been developed to predict drug pharmacokinetics and pharmacodynamics in a faster, more cost-effective, and more accurate manner [181,182].

AI has shown tremendous potential in the fields of pharmacokinetics, pharmacodynamics, and drug discovery [183]. With the advent of powerful computing and machine learning algorithms, AI has emerged as a valuable tool for predicting and optimizing drug pharmacokinetics and pharmacodynamics. Although the challenges of large data and reliable datasets are hard to ignore, AI can open new doors in PKPD studies and their impact on therapies [183,184,185,186,187].

### 6.1. AI-Based Methods to Predict Pharmacokinetic Parameters

**Figure 7:**
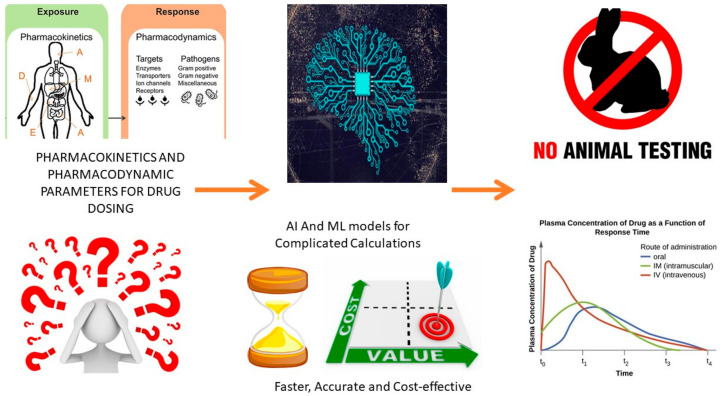
Role of AI in PKPD studies. Pharmacokinetic studies include absorption (A), distribution (D), metabolism (M), and excretion (E) studies. A pharmacodynamic study includes the drug’s effect on the target. Understanding the effect of drug molecules and their distribution requires a large number of calculations. A smaller miscalculation or missed dataset may lead to a huge error that may be critical. AI helps to accelerate complicated calculations without missing datasets and provides more accurate, faster, and cost-effective results. It converts complicated data into easily understandable and representable graphs, which might help to identify the root cause of the problem. It can also help to minimize animal studies by calculating the impact of different conditions such as enzymes, diseased conditions, dosing differences, patient data, etc., in different animals and reduce the number of animals required for clinical trials.

The utilization of machine learning (ML) and deep learning (DL) algorithms is prevalent in the prediction of pharmacokinetic parameters. Various ML algorithms, including the Bayesian model, random forest, support vector machine, artificial neural network, and decision tree, have been employed to forecast drug absorption, distribution, metabolism, and excretion (ADME) characteristics. DL algorithms, including convolutional neural networks (CNNs), long short-term memory (LSTM), and recurrent neural networks (RNNs), are commonly employed in the prediction of various pharmacokinetic parameters, such as drug absorption, bioavailability, clearance, volume of distribution, and half-life. Quantitative structure–activity relationship (QSAR) is a computational approach that utilizes the chemical structure of a molecule to predict its biological activity. This method has found application in pharmacokinetics, where it can be employed to anticipate drug ADME properties, including solubility, permeability, and metabolism (Figure 7) [121,188,189,190,191].

### 6.2. AI-Based Computational Method for PBPK

PBPK models are widely used to simulate drug distribution and clearance in the body. These models are complex, and the development of such models requires extensive data and computational resources. AI-based methods can simplify the development of PBPK models by using machine learning algorithms to identify the most relevant features of the model (Table 4). AI-based computational methods can also optimize the parameters of the PBPK model, which can reduce the need for animal studies and human clinical trials [192,193,194].

The efficacy and safety of drug molecules are largely based on their pharmacokinetic parameters. Drug safety is based on the total time the active drug is present in the body, while the dose of the drug depends on its elimination from the body. Therefore, in vivo exposure is a very important tool for drug safety and efficacy assessment. The drug discovery and development process involves assessment and evaluation prior to clinical trials. Absorption, distribution, metabolism, and elimination (ADME) are the major factors in compound attrition for the development of drug molecules. Drug discovery studies involve in vivo pharmacokinetic studies in animals, while in vitro systems are used for humans along with animal studies. The first, in human dosing, is used for the optimization of the drug’s exposure to humans. In vitro and in vivo extrapolations are used for liver microsomes and hepatocytes. Hepatic clearance is performed with the help of in vivo studies in humans and animals, while in vitro assays are used for liver microsome studies. The human pharmacokinetic parameters are estimated by using allometric scaling methods along with in vivo preclinical data. The volume of distribution, drug clearance, and bioavailability are also estimated by the same method. The simulation of the time course along with ADME properties is simulated by the mathematical framework along with PBPK modeling. The latter are used to understand the in vivo behavior for extrapolation to humans, and normally these are applied to the later stages of drug discovery. The complexity of in vivo data is higher than that of in vitro pharmacokinetic parameters, and AI and ML are implemented for the analysis and assessment of the same [195].

### 6.3. Prediction of Drug Release and Absorption Parameters

AI-based models have been successfully employed to predict drug release and absorption parameters. AI algorithms can analyze data from various drug delivery systems and predict the release kinetics of drugs. By considering factors such as the drug’s physicochemical properties, formulation characteristics, and release mechanism of the delivery system, AI models can estimate the rate and extent of drug release over time. AI-based models can also predict the release kinetics of drugs from different drug delivery systems, such as oral tablets, transdermal patches, and inhalers [196].

AI-based models can predict drug absorption parameters, such as bioavailability and absorption rate, by considering factors such as drug solubility, permeability, and formulation characteristics. These models can analyze the physicochemical properties of the drug, such as lipophilicity and molecular weight, and correlate them with absorption data to estimate how efficiently the drug is absorbed into the bloodstream. Overall, AI-based models provide a powerful tool for predicting drug release and absorption parameters. By analyzing various factors and leveraging machine learning algorithms, these models can optimize drug formulations, guide drug development decisions, and contribute to the design of more effective drug delivery systems [189,190,191,192,193,194,197].

### 6.4. Prediction of Metabolism and Excretion Parameters

AI-based models have proven valuable in predicting drug metabolism and excretion parameters, providing insights into drug pharmacokinetics. AI algorithms can analyze the molecular structure and physicochemical properties of drugs to predict their metabolic pathways. By training on large datasets of known drug metabolism information, AI models can identify structural features associated with specific metabolic transformations. These models enable the prediction of potential metabolites and provide insights into the major enzymes involved in drug metabolism [198].

AI-based models can calculate enzyme kinetics, such as reaction rates and enzyme–substrate interactions, to estimate the metabolic fate of drugs. By considering factors such as enzyme expression levels, genetic variations, and drug–drug interactions, AI models can assess the potential impact of metabolism on drug clearance and efficacy. This information is valuable in optimizing drug dosing regimens and predicting potential drug interactions [199].

AI algorithms can analyze drug physicochemical properties, such as molecular weight, lipophilicity, and ionization, to predict drug clearance rates. By training on datasets that include information on drug clearance pathways, AI models can estimate the rate at which drugs are eliminated from the body. This information is crucial for determining appropriate dosing regimens and ensuring drug efficacy and safety [200].

AI models can predict drug interactions with transporters involved in absorption, distribution, metabolism, and excretion processes. By considering drug physicochemical properties and transporter characteristics, AI models can assess the potential for drug–drug interactions or altered pharmacokinetics due to transporter-mediated effects. This knowledge aids in understanding drug disposition and optimizing drug formulations [201,202,203,204].

By utilizing AI algorithms and analyzing vast amounts of data on drug metabolism and excretion, these models contribute to predicting drug fate in the body. They assist in optimizing drug dosing, identifying potential drug interactions, and aiding in the design of safer and more effective medications. Additionally, AI models enable researchers and pharmaceutical companies to prioritize drug candidates based on their predicted metabolic and excretion profiles, facilitating more efficient drug development processes.

**Table 4:** Algorithms used for the development of AI models for various PKPD studies along with their advantages and limitations.

Algorithm/Software	Aim/Target	Advantage	Limitation	PK/PD/Both	Reference
Bayesian/WinBUGS	To handle data below the limit of quantification	Prior information from the literature can be directly used for model-fitting Easy implementation	Long computational time Negative data in certain PK/PD models which are not possible	Both	[205]
Bayesian/PKBUGS (v 1.1)/WinBUGS (v 1.3)	Pharmacokinetic analysis of sirolimus concentration data for therapeutic drug monitoring	Easy incorporation of prior information with current data Identification of possible covariate relationship	A limited number of datasets and poorly informative data	PK	[206]
Support Vector Machine/Least Square-SVM	Drug concentration analysis of sample drug based on individual patient profile	Personalized model for every new patient SVM-based approaches are more accurate than the PK modeling method for predicting drug concentration	Outliers in samples greatly affect the model, limiting its accuracy	PK	[207]
Support Vector Machine/Drug Administration Decision Support System (DADSS) and Random Sample Consensus RANSAC	Prediction of drug concentration, ideal dose, and dose intervals for a new patient	More flexible and structurally adjustable	The noise of datasets impacts the overall predictability of the algorithm	PK	[208]
Support Vector Machine/Profile Dependent SVM	Therapeutic drug monitoring of kidney transplant recipient	Critical dosing and cost-effective Effective for nonlinear models	Time-consuming Large datasets	PK	[209]
Support Vector System + Random Forrest Model	Pharmacodynamic drug interaction based on Side-Effect Similarity (SES), Chemical Similarity (CS), and Target Protein Connectedness (TPC)	PDI was predicted with an accuracy of 89.93% and an AUC value of 79.96%	Requires larger data processing and filtration	PD	[210]
Linear Regressions (LASSO)/Gradient Boosting Machines/XGBoost/Random Forest	Prediction of the plasma concentration–time series and area under the concentration-versus-time curve from 0 to 24 h after repeated dosing of Rifampicin	Time-efficient analysis Improves method for covariate selections	Risk of results being not clinically relevant	PK	[182]
XGBoost	Estimation of drug area under the curve (AUC) of tacrolimus or mycophenolate mofetil (MMF)	Pharmacokinetic (PK) datasets from renal, liver, and heart transplant patients were predicted accurately	Not possible to calculate the probability of target attainment and accurate dosing	PK	[211,212]
Simulated Annealing k-Nearest-Neighbor (SA-kNN)/Partial Least-Square (PLS)/Multiple Linear Regression (MLR)/Sybyl version 6.7	Prediction of pharmacokinetic parameters of antimicrobial agents in humans based on their molecular structure	Cost-effective Requires less sample size	Requires multiple model generation methods Interpretation of individual descriptors is almost impossible	Both	[213]
Drug Target Interaction Convolutional Neural Network (DTICNN)	Identification of the drug-target interactions and predict potential drug molecules	Cost-effective Time-saving	Large datasets are required	PD	[214]
Deep Long Short-Term Memory (DeepLSTM)	Computational methods to validate the interaction between drugs and target	Based on Position Specific Scoring Matrix (PSSM) and Legendre Moment (LM) (drug molecular substructure fingerprints)	Large datasets are required	PD	[215]

## 7. Limitations of AI Tools

Despite their benefits, AI-based models have some limitations, such as the need for large datasets, potential biases, and lack of interpretability. Therefore, AI-based models should be used in combination with traditional experimental methods to ensure the safety and efficacy of drugs. Some of the limitations are highlighted below:

### 7.1. Lack of Transparency

AI models use complex algorithms and are often referred to as “black boxes” because it is difficult to understand how the model arrives at its predictions. This lack of transparency can make it challenging to gain regulatory approval for AI-based drug development tools, as it can be challenging to demonstrate that the model is making accurate and reliable predictions. Furthermore, the lack of transparency can also lead to a lack of trust in the model’s predictions, particularly if the model makes predictions that conflict with the expectations of clinicians or researchers [216,217].

### 7.2. Limited Availability of Data

AI models require a significant amount of data for accurate predictions. However, in some cases, there may be limited data available for a particular drug or population, leading to less accurate predictions or biased results. For instance, rare diseases may have limited data available, which can be a significant challenge for developing AI models. Additionally, the data used to train AI models may not be representative of the population of interest, which can lead to biased results. Moreover, some types of data, such as longitudinal data or real-world evidence, may not be readily available, which can limit the utility of AI models. These limitations highlight the need for the careful consideration of the quality and representativeness of the data used to develop AI models.

### 7.3. Biases in Data

The efficacy and precision of AI models are contingent upon the quality of the data utilized for their training. In instances where the data exhibit bias or incompleteness, the resulting predictions may also be biased. The homogeneity of patient populations in clinical trials is a significant problem within the realm of pharmacology. If a specific demographic or disease state is inadequately represented in the training dataset, the model’s ability to make precise predictions regarding the drug’s efficacy in that particular population may be compromised. Moreover, in the case of incomplete or inaccurate data, the model may generate erroneous assumptions, which can result in imprecise predictions. The utilization of an AI model to direct clinical decision-making can pose a significant challenge. Therefore, it is essential to guarantee that the training data used to create AI models are representative of the population for whom the model will be utilized and that the data are trustworthy, comprehensive, and impartial [218,219].

### 7.4. Inability to Incorporate New Data

Once an AI model is trained, it is often challenging to incorporate new data or update the model. This can be a significant limitation in the context of drug development processes, where new information and data are constantly emerging. For example, as new drugs are introduced or as clinical trials produce additional data, an AI model may need to be updated to reflect this new information. However, updating an AI model can be challenging, and it may require significant time and resources to retrain the model with the new data. Furthermore, as drug development processes continue to evolve, AI models must be able to keep up with these changes. Failure to do so could result in inaccurate predictions and flawed decision-making. Thus, it is crucial to carefully consider the limitations of AI models and to develop strategies for updating them as new information becomes available. This can include designing models that can be easily updated or integrating the model into a larger framework that can be continuously refined over time.

### 7.5. Limited Ability to Account for Variability

AI models are generally trained on large datasets, which can be biased toward the average responses observed in the data. As a result, the models may not be able to accurately predict drug responses for individuals who deviate significantly from the average response. This is particularly concerning for drugs that have a wide range of responses in different patients (such as in cancer), where the variability can be significant [220].

### 7.6. Interpretation of Results

AI models can be complex and can generate outputs that are difficult to interpret, even for experts in the field. The models may not be able to provide a clear explanation of how they arrived at their predictions, which can make it challenging for clinicians and researchers to understand and interpret the results. In some cases, the results may be difficult to translate into actionable insights that can be used in clinical practice or drug development. Additionally, the use of AI models may require a level of technical expertise that is not readily available to all clinicians and researchers, which can further limit their usefulness. As a result, there is a need for an improved interpretability and explainability of AI models, to ensure that their predictions can be understood and used effectively [221,222].

### 7.7. Ethical Considerations

As with any use of AI, there are ethical considerations that must be taken into account when using these technologies in drug development. One major concern is patient privacy, as sensitive health data are often used to train AI models. Data safety and security represent crucial parameters that demand significant attention and cannot be overlooked. It is important to ensure that patient data are collected and used in a way that protects their privacy and respects their rights. Data ownership is another ethical concern when using AI in drug development. In some cases, data may be collected from patients without their explicit consent, and it may not be clear who owns the data or who has the right to use it. This can lead to conflicts between patients, researchers, and pharmaceutical companies [223,224]. Regulatory agencies are tasked with the development of stringent protocols, guidelines, and standardized evaluation processes to effectively integrate AI into drug development. These measures should encompass multiple dimensions, including the ethical considerations of animal welfare and patient safety. Animal testing, which plays a pivotal role in drug development, necessitates a commitment to reducing, refining, and replacing animal models whenever feasible, aligning with ethical principles. Prioritizing patient safety, AI models must undergo thorough validation and testing to ensure their reliability and accuracy. An important step in addressing the regulatory and ethical implications of AI in drug development is the release of the discussion paper by the U.S. Food and Drug Administration (FDA) entitled, “Using Artificial Intelligence & Machine Learning in the Development of Drug and Biological Products.” This document provides an overview of the role of AI in drug discovery, nonclinical research, and clinical research. Additionally, it outlines recommended practices for the application of AI and machine learning. This FDA initiative marks an important milestone in regulating the use of AI in healthcare and paves the way for new opportunities in the sector. It signifies the recognition of the potential benefits and challenges associated with AI in drug development and sets the stage for future regulatory advancements in this domain [225].

### 7.8. Complex Biological Systems

AI’s ability to accurately mimic the complexity of biological systems as a whole is limited. Biological systems are intricate and dynamic, encompassing a multitude of interconnected pathways, feedback loops, and intricate molecular interactions. This complexity poses challenges for AI models, which often simplify and abstract the underlying biological processes. AI models heavily rely on training data to learn patterns and make predictions, but the available data may not fully capture the intricacies and nuances of biological systems [226]. Factors such as genetic variations, environmental conditions, and interindividual variability contribute to significant complexity and variability that may not be adequately captured by AI models [45,227]. Moreover, the emergent properties of biological systems, where the collective behavior of individual components gives rise to system-level behaviors, are difficult to predict solely based on the properties of individual components. A limited understanding of certain biological processes and mechanisms further hampers the accurate incorporation of this knowledge into AI models [228].

### 7.9. Lack of Clinical Expertise

While AI can identify correlations, it is essential to recognize that individual patient therapies can vary despite these correlations. AI algorithms typically operate on a statistical framework, which may limit their comprehension of the intricate factors and the profound effects certain parameters can have. The complex nature, where treatment decisions are influenced by various individualized factors, poses a challenge for AI models primarily focused on statistical associations [229]. Therefore, the ability of AI to fully capture the critical aspects and implications of specific parameters may be limited.

### 7.10. Inactive Molecules

AI uses a computational approach to predict the binding interactions between a small molecule and a target protein by employing algorithms and scoring functions. However, such simulations can lead to the identification of inactive molecules. One major challenge is accurately representing the conformational flexibility of both the small molecule and the target protein, as docking algorithms sample a limited range of conformations, potentially resulting in false-positive or false-negative binding affinities [34]. Moreover, if the protein structure used in docking or AI is incomplete or inaccurate, it can lead to erroneous predictions. Difficulties in accounting for solvation effects, receptor flexibility, and other influential factors further contribute to the limitations of docking. Hence, it is crucial to conduct experimental validation to confirm the activity of identified compounds, assessing their potency and selectivity. Continuous efforts to refine docking algorithms, scoring functions, and incorporate factors such as protein flexibility and solvent effects aim to enhance the reliability of docking-based screening. Integrating additional computational methods, such as molecular dynamics simulations, can provide a more comprehensive representation of molecular interactions [230].

Despite the limitations of AI tools, they hold significant potential and cannot be overlooked in the field of pharmaceutical development. It is crucial to promptly identify and address these limitations to facilitate smoother and faster advancements in the industry. Recent years have witnessed rapid progress in resolving these challenges, driven by improvements in data availability, deep learning algorithms, explainability, integration with other modeling approaches, and increased computational power [231].

However, one persistent problem that remains unresolved is the issue of misreported data, which introduces bias and distorts the accuracy of AI models. To mitigate this, it is imperative to adopt the principles of FAIR data (Findable, Accessible, Interoperable, Reusable), which align with the fundamental principles of ALCOA (Attributable, Legible, Contemporaneous, Original, and Accurate) [232]. By adhering to these principles, data quality can be improved, enhancing the reliability of AI-driven analyses.

While challenges associated with AI in PKPD studies are substantial and will require time to overcome, the ever-evolving nature of the field instils hope for continuous improvement. However, it is crucial to exercise caution and not overly rely on AI without considering potential limitations and verifying results through rigorous scientific validation. Although AI has shown great potential in improving and enhancing PKPD studies, it is not ready to completely replace humans in this field. AI is a powerful tool that can assist researchers and clinicians in analyzing large amounts of data, identifying patterns, and making predictions. While AI can automate certain tasks and assist in data analysis, the collaborative effort between AI and human experts is crucial for successful PKPD studies. The integration of AI in pharmaceutical research should be approached with a balanced perspective, acknowledging both its potential and the need for careful evaluation and validation.

## 8. Current Trend: Fairy Tale to the Holy Grail

The current trends demonstrate the wide-ranging impact of AI in pharmaceutics, spanning drug discovery, precision medicine, formulation optimization, clinical trials, safety monitoring, and supply chain management. Here are some prominent trends:

- Drug Discovery and Development: AI is revolutionizing the drug discovery process by enabling virtual screening, molecular modeling, and predictive analytics. AI algorithms can analyze vast amounts of chemical and biological data to identify potential drug candidates, optimize lead compounds, and predict their properties. This expedites the identification and development of novel therapeutics.
- Precision Medicine: AI is being utilized to advance precision medicine approaches. By analyzing patient data, including genomics, proteomics, and clinical records, AI algorithms can identify patient subgroups, predict treatment responses, and assist in personalized treatment decision-making. AI also contributes to the development of biomarkers for disease diagnosis and prognosis.
- Drug Repurposing: AI is being applied to identify new uses for existing drugs, a process known as drug repurposing. By analyzing large datasets and biological knowledge, AI algorithms can identify potential drug–disease associations and repurpose approved drugs for new therapeutic indications. This approach offers a faster and more cost-effective route to drug development.
- Drug Formulation and Delivery: AI plays a role in optimizing drug formulations and delivery systems. AI models can predict drug release kinetics and absorption profiles and optimize formulations for enhanced efficacy and targeted delivery. AI is also used to design drug delivery devices and systems that improve patient adherence and convenience.
- Clinical Trial Optimization: AI is being leveraged to optimize clinical trials, improving efficiency and reducing costs. AI algorithms can aid in patient recruitment, identify suitable trial populations, and optimize trial protocols. AI also assists in the real-time monitoring and analysis of trial data, allowing for adaptive trial designs and faster decision-making.
- Regulatory Compliance and Safety: AI is increasingly used to support regulatory compliance and ensure drug safety. AI algorithms can analyze real-world data, adverse event reports, and the literature to identify potential safety issues and monitor post-marketing drug safety. AI also helps in pharmacovigilance, signal detection, and adverse event prediction.
- Supply Chain Optimization: AI is applied to optimize pharmaceutical supply chains, ensuring efficient manufacturing, inventory management, and distribution. AI algorithms can predict demand, optimize production schedules, and enhance quality control processes, contributing to more streamlined and cost-effective operations.

Pharmaceutical companies are increasingly recognizing the potential of AI in PKPD studies. AI offers valuable tools and approaches that can enhance drug discovery and development processes. These companies are leveraging AI to analyze large datasets, predict drug–target interactions, optimize drug candidates, and simulate drug responses in biological systems. Some examples include GNS Healthcare [233], AstraZeneca [234], Atomwise [235], Recursion Pharmaceuticals, and Insilico Medicines [236]. AI has helped to improvise strategies for rapid and more accurate dosage form development. Pfizer has utilized AI algorithms to predict drug–drug interactions (DDIs) by analyzing vast datasets of drug structures, clinical outcomes, and adverse effects [237]. This approach has enabled Pfizer to identify potential DDIs more efficiently and prioritize drug combinations for further investigation, minimizing the risk of adverse reactions. Novartis has leveraged AI in drug formulation and delivery optimization, employing algorithms to analyze physicochemical properties, solubility, and permeability data to design optimal drug formulations and delivery systems. This has streamlined the drug development process and improved bioavailability and therapeutic efficacy. Additionally, Roche has made significant strides in personalized medicine by integrating patient-specific data into AI models [238]. By incorporating genetic profiles, medical histories, and biomarker measurements, Roche can predict individual drug responses and tailor treatment regimens, leading to more effective and personalized therapies. These examples highlight the innovative use of AI by pharmaceutical companies and showcase how it has revolutionized PKPD studies, paving the way for enhanced drug development strategies and improved patient outcomes. Some of the major applications of AI in pharmaceutical companies are tabulated in Table 5.

## 9. Futuristic Overview

AI might revolutionize the pharmaceutical industry in the future to accelerate drug discovery and drug development. Virtual screening techniques will rapidly analyze enormous chemical libraries and find therapeutic candidates with required features, accelerating lead compound identification. AI-enabled precise medicine could categorize patients, predict therapy responses, and customize medicines by analyzing genomes, proteomes, and clinical records. Scientists may create innovative compounds with target-binding characteristics using deep learning and generative models, improving medication effectiveness and lowering adverse effects. Additionally, AI will allow patient-specific dose formulations. AI algorithms will optimize medicine compositions and delivery methods to improve treatment results by considering patient-specific parameters, including age, weight, genetics, and illness status. AI algorithms will revolutionize safety assessment by predicting drug candidate side effects and toxicity.

AI-powered monitoring systems will allow remote patient care and medication adherence. Wearable gadgets and sensors will continuously gather data for AI algorithms to propose personalized therapy and better compliance. AI improves clinical trial design, patient selection, and recruitment. AI algorithms will use electronic health records, biomarkers, and genetic profiles to find appropriate patients, lower trial costs, and speed up approval.

The real-time monitoring and control of important parameters by AI models will optimize continuous manufacturing operations. AI algorithms will make pharmaceutical manufacture uniform and efficient via data analysis and feedback. AI will analyze large amounts of data to inform regulatory decisions. It will assist regulatory bodies in speeding up medication approval and improving safety.

The use of artificial intelligence in various segments of healthcare is growing daily, from the triage and screening of clinical risk prediction to diagnosis [141,240]. Clinical applications of AI have the potential to increase diagnosis accuracy and healthcare efficiency. The massive amount of time and money spent on medication research and development necessitates the use of more inventive methodologies and tactics [241]. Artificial intelligence is providing large opportunities in the medical field, such as multivariate data analysis of abundant amounts; resolving the complicated issues involved in the creation of viable medication delivery systems; making decisions with more accuracy, disease categorization, and modeling; establishing the correlation between formulations and processing factors; dosage ratio optimization; rapid drug development; anticipating drug bioactivities and interactions; cellular response; the effectiveness of the drugs used in combination; the outcomes of treatment; and many more. As demonstrated in all sections, AI and machine learning have considerable potential in revolutionizing medication delivery to improve infectious disease treatment effectiveness. Unfortunately, there are currently limited practical uses of AI in medication delivery, particularly in the therapeutic setting. Various AI methods used in drug delivery for the treatment of infectious diseases, such as Boost, 𝑘-nearest neighbors, decision trees and random forest, Naïve Bayes, ANN, Feedback System Control (FSC), SVM, Set Covering Machine (SCM), and logistic regression, have not been widely evaluated or used in clinical settings, demonstrating the existence of significant hurdles in the clinical translation of AI for medication administration in the treatment of infectious diseases [96,144]. Machine learning and artificial intelligence combined with PBPK modeling are important tools for drug development and risk assessment of environmental chemicals. A recently developed model of PBPK was used to describe how chemicals enter the body, the bioavailability of drugs, the movement of drugs between tissues, and how drugs are metabolized and eliminated from the body by a mathematical description. For the determination of the toxicity of the various classes of nanomaterials, PBPK-based toxicity models are most suitable. Because the chemical ADME routes are not well described or mathematically formulized, developing mechanistically valid PBPK models for novel compounds with limited prior knowledge is difficult and complex. With the recent development of Neural-ODE (Neural-ordinary differential equation) algorithms, it is now feasible to build PBPK simulations for a novel medication based on its properties, which can learn the governing ODE equations algorithmically and directly from PK data without the need for well-characterized previous knowledge. Overall, advances in AI approaches, particularly for the deep neural network model, may help to solve some of today’s challenges, thereby improving the performance of PK and PBPK modeling and simulations aimed at drug discovery and development, as well as a human health risk assessment of environmental chemicals [242]. The ultimate goal of the development of AI in PKPD depends on the understanding of the fundamentals associated with different scientific principles. This is only possible by developing standard regulations with strict measures that prevent the abuse of AI but at the same time accelerate its growth. Such a tedious task requires the collaboration of multiple pharmaceutical companies and regulatory bodies along with various healthcare professionals, including doctors, nurses, pharmacists, data scientists, etc.

While this futuristic overview presents exciting possibilities, it is important to recognize that challenges related to data quality, regulatory frameworks, and ethical guidelines will need to be addressed for the full realization of AI’s potential in pharmaceutical product development. However, with continued advancements and collaborations between industry, academia, and regulatory bodies, AI-driven innovations have the potential to revolutionize the pharmaceutical industry and improve patient outcomes in the years to come.

## 10. Conclusions

AI is transforming drug delivery technologies, enabling targeted, personalized, and adaptive therapies. By leveraging AI’s capabilities in data analysis, pattern recognition, and optimization, pharmaceutical researchers and healthcare professionals can enhance drug efficacy, minimize side effects, and improve patient outcomes. AI-based methods have revolutionized the field of pharmacokinetics and pharmacodynamics. They offer several advantages over traditional experimental methods. AI-based models can predict pharmacokinetic parameters, simulate drug distribution and clearance in the body, and optimize drug dosage and administration routes. AI-based computational methods for PBPK models can simplify the development of such models and optimize their parameters, reducing the need for animal studies and human clinical trials. Computational pharmaceutics, facilitated by AI and big data, revolutionizes the drug delivery process by providing a more efficient, cost-effective, and data-driven approach. It enables the optimization of drug formulations, personalized therapies, regulatory compliance, and risk reduction, ultimately leading to improved drug manufacturing processes and enhanced patient outcomes. Overall, the integration of AI technologies holds great promise for accelerating drug development, improving patient outcomes, and revolutionizing the pharmaceutical industry, promoting its evolution from era 4.0 to era 5.0.

## Figures and Tables

**Table 2:** Popular AI model tools used for drug discovery.

AI Model Tools	Summary
DeepChem	An open-source library that provides a wide range of tools and models for drug discovery, including deep learning models for molecular property prediction, virtual screening, and generative chemistry.
RDKit	A widely used open-source cheminformatics library that offers various functionalities for molecule handling, substructure searching, and descriptor calculation. It can be integrated with machine learning frameworks for drug discovery applications.
ChemBERTa	A language model specifically designed for drug discovery tasks. It is based on the Transformer architecture and is pretrained on a large corpus of the chemical and biomedical literature, allowing it to generate molecular structures, predict properties, and assist in lead optimization.
GraphConv	A deep learning model architecture that operates on molecular graphs. It has been successful in predicting molecular properties, such as bioactivity and toxicity, by leveraging the structural information encoded in the graph representation of molecules.
AutoDock Vina	A popular docking software that uses machine learning techniques to predict the binding affinity between small molecules and protein targets. It can assist in virtual screening and lead optimization for drug discovery.
SMILES Transformer	A deep learning model that takes Simplified Molecular Input Line Entry System (SMILES) strings as input and generates molecular structures. It can be used for de novo drug design and lead optimization.
Schrödinger Suite	A comprehensive software package for drug discovery that incorporates various AI-driven tools. It includes modules for molecular modeling, virtual screening, ligand-based and structure-based drug design, and predictive modeling.
IBM RXN for Chemistry	An AI model designed to predict chemical reactions. It utilizes deep learning algorithms and large reaction datasets to generate potential reaction outcomes, aiding in the discovery of new synthetic routes and compound synthesis.
scape-DB	scape-DB (Extraction of Chemical and Physical Properties from the Literature-DrugBank) is a database that combines natural language processing and machine learning to extract chemical and biological data from the scientific literature. It provides valuable information for drug discovery research.
GENTRL (Generative Tensorial Reinforcement Learning)	A deep learning model that combines reinforcement learning with generative chemistry to design novel molecules with desired properties. It has been used for de novo drug design and optimization.

**Table 5:** Lit of companies using AI and ML technologies in pharmaceutical research [239].

Sr. No.	Domain	Technology and Outcome	Industry and Collaborations
1	Drug design	Novel therapeutic antibodies	Exscientia
2	Molecular drug discovery	AtomNet–deep learning-driven computational platform for structure-based drug design	AtomWise
3	Gene mutation related disease	Machine learning based recursion operating system for biological and chemical datasets	Recursion
4	Drug design	Ligand- and structure-based de novo drug design, especially in multiparametric optimization	Iktos
5	Drug discovery	Generative modeling AI technology	Iktos and Galapagos
6	Drug development	Potential preclinical candidates	Iktos and Ono Pharma
7	Drug design	Rapid drug design by software “Makya”	Iktos and Sygnature Discovery
8	Drug discovery and Drug development	Pharma.AI, PandaMics, ALS.AI	Insilico Medicine
9	Drug target and Drug development	ChatPandaGPT	Insilico Medicine
10	Drug development	Protein motion in drug development lie RLY-4008 (Novel allosteric, pan mutant and isoform selective inhibitor of PI3Kα	Relay therapeutics
11	Drug discovery	AI and machine learning for selection of drug target	BenevolentAI
12	Drug target	Drug target selection for chronic kidney disease and idiopathic pulmonary fibrosis	BenevolentAI and AstraZeneca, GlaxoSmithKline, Pfizer
13	Clinical trials	AI in clinical trials	Pfizer and Vysioneer
14	Disease treatment	AI and supercomputing for oral COVID-19 treatment Paxloid	Pfizer
15	Drug discovery	NASH drugs and sequencing behemoth Illumina	AstraZeneca and Viking therapeutics
16	Drug development	Trials360.ai platform in clinical trials for site feasibility, site engagement and patient recruitment	Janssen
17	Drug research	Automate medical literature review by using natural language processing	Sanofi
18	Drug development	AI in drug development	BioMed X and Sanofi
19	Drug research and drug development	AI empowerment and AI exploration platforms	Novartis and Microsoft
20	Drug discovery	AI drug discovery platform	Bayer

## Data Availability

No new data were created or analyzed in this study. Data sharing is not applicable to this article.
